# A factor confirmation and convergent validity of the “areas of worklife scale” (AWS) to Spanish translation

**DOI:** 10.1186/1477-7525-11-63

**Published:** 2013-04-18

**Authors:** Santiago Gascón, Michael P Leiter, Naomi Stright, Miguel A Santed, Jesús Montero-Marín, Eva Andrés, Angela Asensio-Martínez, Javier García-Campayo

**Affiliations:** 1 University of Zaragoza, Faculty of Psychology, Zaragoza, Spain; 2 Centre for Organizational Research & Development, Acadia University, Acadia, Canada; 3 UNED, Faculty of Psychology, Madrid, Spain; 4 University of Zaragoza, Faculty of Medicine, Zaragoza, Spain; 5 REDIAPP “Network on Preventive Activities and Health Promotion, Barcelona, RD06/0018/0017, Spain; 6 Unit of Clinical Research “12 de Octubre Hospital, Madrid, Spain; 7 CIBER-Epidemilogy & Health Public, Madrid, Spain

**Keywords:** Stress, Burnout measure, Areas of worklife

## Abstract

**Background:**

Perceived incongruity between the individual and the job on work-life areas such as workload, control, reward, fairness, community and values have implications for the dimensions of burnout syndrome. The “Areas of Work-life Scale” (AWS) is a practical instrument to measure employees´ perceptions of their work environments.

AIMS: Validate a Spanish translation of the AWS, and it relationship with Masclach Burnout Inventory dimensions.

**Methods:**

The study was conducted in three medium-sized hospitals and seven rural and urban Primary Care centres (N = 871) in Spain*.* The “Maslach Burnout Inventory General Survey” (MBI-GS) and AWS were applied. We developed a complete psychometric analysis of its reliability, and validity.

**Results:**

Data on the reliability supported a good internal consistency (Cronbach α between .71, and .85). Construct validity was confirmed by a six factor model of the AWS as a good measure of work environments (χ^2^_(352)_ = 806.21, *p* < 0.001; χ^2^/df = 2.29; CFI = 0.935, RMSEA = 0.039); concurrent validity was analysed for its relationship with other measures (opposing dimensions to burnout, and MBI), and each correlation between dimensions and sub-dimensions were statistically significant; as well, predictive validity, by a series of Multiple Regression Analysis examined the resulting patterns of the Confirmatory Factor Analysis (CFA) confirms the relationship between the work-life areas and burnout dimensions.

**Conclusions:**

Leiter and Maslach’s AWS has been an important instrument in exploring several work-life factors that contribute to burnout. This scale can now be used to assess the quality of work-life in order to design and assess the need for intervention programs in Spanish-speaking countries.

## Background

In Spain and Spanish-speaking countries, burnout is rarely diagnosed as a psychological disorder. Instead, doctors, psychiatrists, and psychologists diagnose depression, anxiety or adaptive disorder due to work stress. This results in burnout being easily confused with those disorders as professionals do not know the true dimensions of burnout, and lack adequate diagnostics tools [1]. Although burnout is not recognized as an occupational disease, in Spain many courts pass judgments which consider it to be a workplace accident [2]. The Spanish Legislation [3] requires organizations to undertake an evaluation of psychosocial risks, but these laws have limited impact, partially due to inconsistent enforcement of the regulations [4].

The term “burnout” has been used to describe a fundamental disconnection between employees and the workplace, and to describe an experience of exhaustion [5,6]. But the syndrome is more complex [7].

In 1981, Maslach and Jackson developed the Maslach Burnout Inventory (MBI) [8]: A short questionnaire that approaches this syndrome from three dimensions – Emotional Exhaustion, Depersonalization and Personal Accomplishment –. Since then, this diagnostic tool has been used to study various professional groups around the world [1]. The authors understand burnout to be the most serious consequence of job stress, when all coping strategies have failed and the individual feels emotionally drained, unconnected to their work and useless. The authors don’t regard burnout as a question of “*all or nothing”*, but consider each individual to be situated at some point along these three dimensions -over time feeling more fulfilled and involved, or the opposite, more exhausted.

While the questionnaire has demonstrated its usefulness when it comes to measuring the dimensions of burnout, it doesn’t provide information about areas in work-life that may contribute to a possible burnout. For this reason, Leiter and Maslach [9] created the questionnaire “Areas of Work-life Scale” (AWS) [10], which measures both the opposing dimensions of burnout -Energy, Implication and Efficacy- and the areas of work that could contribute positively or negatively to these three dimensions.

We consider this tool to be of great interest in order to design effective intervention programs, as it highlights which aspects of the organization should be acted on. AWS is not a tool to measure burnout individually (although it can be used in this manner), as the authors consider burnout to be a problem that the individual cannot cope with alone. The questionnaire regards the organization as a subject for evaluation and intervention, since organizational aspects of work-life (overload, control, reward, community feeling, fairness, and values) contribute to employees feeling energetic and involved in their tasks, or the contrary.

Perceived incongruity between the individual and the job regarding the six areas of work-life has been shown to detect burnout [11].

The aims of the study were to confirm the structure of the six factors in the Spanish translation of the AWS, and its implications on the three dimensions: Exhaustion, Depersonalization and Inefficacy, in order to have an effective and practical instrument to measure employees´ perceptions of their work environments [9].

The measure of MBI is already well established in the research literature (also in Spanish) as having a consistent factor structure and stability over time [12-14].

Using their AWS, Leiter and Maslach have explored a two-process model of burnout [15,16] not only based on exhaustion by overload, but also based on personal and organizational value conflicts. As well as the “exhaustion”, “depersonalization” and “cynicism” dimensions, the focus from individuals’ concern with physical or emotional wellbeing to considering their capacity to connect with the external world is expanded on. The third dimension, “Efficacy”, describes employees’ self evaluations. The experience of chronic exhaustion and cynicism erodes employees’ belief in their capacity to exert influence over their work environment [11,15]. In light of much research, Leiter and Maslach [9-11] predicted that value incongruity has implications for all three aspects of burnout: people start a job with enthusiasm and expectations of success. Over time, employees conclude that there are areas of work-life that are in conflict with their needs. As a result of poor person/job fit, employees become exhausted, frustrated, cynical, and discouraged [17].

Burnout has been recognized as an important personal and social problem: while some employees may quit their job, others will stay on doing only the bare minimum rather than their very best. In addition to the problems of health disorders, absenteeism and loss of work hours, a dramatic outcome of burnout is the definitive resignation of qualified employees, which is detrimental to the organization [9,17].

The goal of Leiter and Maslach’s research has been to design tools for both researchers and practitioners [9-11]. They have developed a new model that draws on the extensive research literature on job stress and proposes that six areas of job-person mismatch are the critical sources of burnout [9,17].

**Figure 1:**
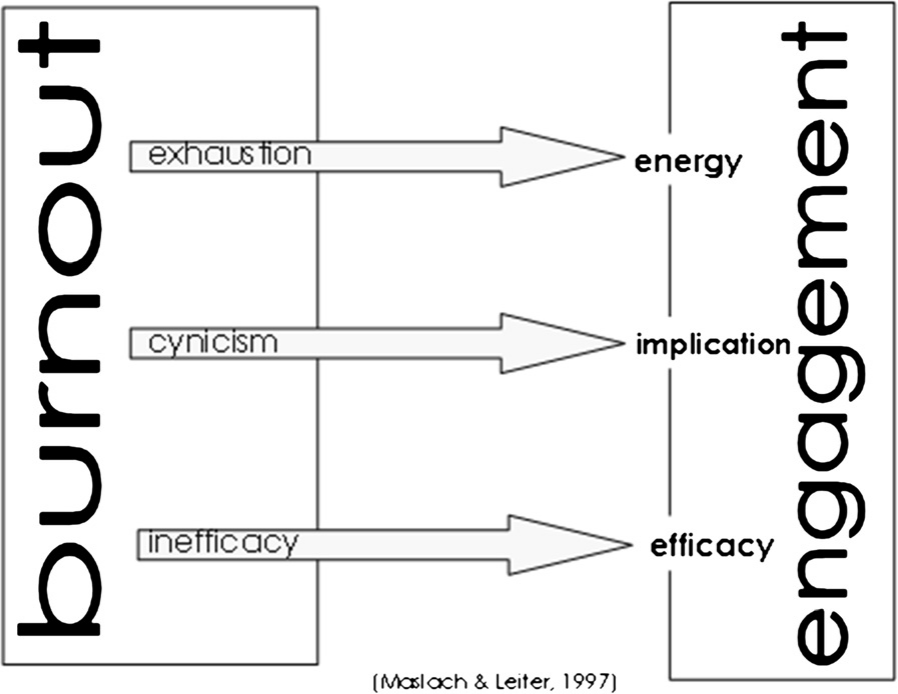
Burnout – engagement dimensions.

An important characteristic of this model is the concept of burnout as a continuum in the relationship people establish with their jobs. In contrast to a syndrome of Exhaustion, Implication/Cynicism and Inefficacy, Leiter and Maslach proposed a positive state of Energy, Involvement, and Efficacy [17]. They defined engagement on the same dimensions as burnout, but placed it on the positive end of these three qualities. Thus, engagement comprises a state of high energy, strong involvement, and a sense of efficacy (Figure 1).

### Six areas of worklife

In studies of job stress, the authors have identified six key dimensions: workload, control, reward, fairness, community, and values [11,17]. The first two areas, Workload and Control, reflect the Demand-Control model of job stress [18,19]. Reward refers to the power of reinforcement to shape behavior [20,21]. Community reflects on social support and interpersonal conflict [22,23], while fairness comes from literature on equity and social justice. Finally, value reflects the cognitive-emotional power of job goals and expectations [24].

A critical aspect throughout the literature on burnout is the problematic relationship between employees and their environment, which is often described in terms of imbalance: the demands of the job exceed their capacity to cope effectively, or an individual’s efforts are not reciprocated with equitable rewards [22]. There is a long trajectory within psychology, explaining behavior in terms of the interaction of an individual and their environment: personality and vocational psychology; individual-environmental congruence [25]. Leiter and Maslach extend the job-person paradigm to a broader and more complex conceptualization of individuals in their job context, in a model that focuses on the degree of experienced congruence between the individual and the six dimensions of the individual’s job environment [11]. They propose that the greater the perceived gap between the individual and the job, the greater the likelihood of burnout and conversely, the greater the consistency, the greater the likelihood of engagement with work.

The goal of these authors was to develop a measure that would apply the concept of job-person fit to the assessment of the six key areas of worklife, in a simple format that could be utilized by a wide range of employees. This new measure has the potential to provide useful diagnostic information to organizations interested in intervention [9].

## Methods

### Participants answered the following questionnaires

- A Demographic Data Record which asked for personal, family and workplace information.

– *Maslach Burnout Inventory-General Survey* (MBI). Developed from the original MBI [8,14] the 22 items are framed as statements of job-related feelings (e.g., “I feel burned out by my work”; “I feel confident that I am effective at getting things done”) and are rated on a seven-point frequency scale (ranging from 0 = “never” to 6 = “daily”). The questionnaire uses a seven-point Likert response scale and evaluates three burnout domains: Emotional Exhaustion (EE) by means of nine items: Depersonalization (DP: five items) and Personal Accomplishment (PA: eight items). These dimensions are calculated as the sum of corresponding items. Each MBI domain has cut off points established by Maslach [8]: for EE ≥27, DP ≥10 and PA ≤10. Burnout is reflected by higher scores on exhaustion and depersonalization or cynicism, and lower scores on personal accomplishment or efficacy [11]. The questionnaire is valid for the Spanish population [14]. Cronbach’s alpha for the MBI sub-scales emotional exhaustion, depersonalization, and personal accomplishment were: 0.67, 0.66 and 0.70 respectively. VARIMAX rotations were undertaken to examine the inter-relationship of different items.

The psychometric properties of the Spanish version have been studied meanwhile an exploratory factor analysis [14].

– *Areas of Work-life Scale*[9,11,12]: This questionnaire is comprised of 45 items:

a) A first questionnaire of 16 items that provide information on the three dimensions opposite to burnout: Energy (at the opposite end to the exhaustion), Efficacy -opposite to depersonalization or cynicism-, and Implication -or personal accomplishment- This first subscale has showed high correlation with MBI dimensions and adequate psychometric properties both in English and Spanish and

b) A second questionnaire that is made up of six sub-scales –or areas- (29 items) that produce distinct scores of perceived congruence or incongruence between the individual and the job for each area: workload, control, reward, community, fairness and values (as already explained in the previous section). Each sub-scale includes positively worded items of congruence, e.g., “I have enough time to do what’s important in my job” (manageable workload) and negatively worded items of incongruence, e.g., “Working here forces me to compromise my values” (values). Respondents indicate their degree of agreement with these statements on a 5-point Likert-type scale ranging from 1 (strongly disagree) to 5 (strongly agree). The scoring for the negatively worded items is reversed. For each of the six sub-scales, the AWS measure defines congruence as a high score (greater than 3.00), indicating a higher degree of perceived alignment between the workplace and the respondent’s preferences. Conversely, it defines incongruence as a low score (less than 3.00), indicating more perceived misalignment or misfit between the employee and the workplace.

The AWS has yielded a consistent factor structure across samples (n = 8,339) from different organizations [11]. The alpha values for all scales meet the 0.70 criterion. An anonymous code was created for each participant.

### Procedure

For the Spanish translation of the questionnaire followed the protocol poposed by the International Quality of Life Assessment (IQQLA) [26]. The original questionnaire was translate into Spanish by two bilingual research team members whose first language was Spanish. Each one made an independent translation of the questionnaire items and response option. Subsequently, the translators met with the principal investigator to agree on a common translations, discuss the differences, and alternatives decisions were made according to three criteria: clarity, use of common language, and conceptual equivalence. Preliminary translation was again reviewed by two interpreters, whose mother language was English. They got a new English version (back translation), which was compared with the original version to assess the conceptual equivalence. Finally, we conducted a pilot study using the questionnaire obtained in this second review in order to assess the understanding and the feasibility of administering the questionnaire, as well as gauge the response options to a sample of 112 participants. After conducting a factor analysis to verify its structure, the questionnaire had the same dimensions as the originals, however there were several items than were not associated with any dimension, due not only to translation problems, but to conceptual and cultural differences regarding work-life. The team re-wrote these items, respecting their original concepts and altered them to reflect Spanish organizational culture.

### Sample size

The sample consisted of 1200 health care workers, who had remained in the current job at least a year, excluding resident workers. This group was made up of 60,7% nurses and 39,2% doctors. The average age of participants was 41.84 years (S.D. 8.427). 64.2% were women and 35.8% men. Selected from three medium-sized hospitals and seven rural and urban Primary Care centers (N = 871) in Spain. Using for centre selection, criteria of representativeness regarding the most important variables.

The questionnaires were distributed in informative sessions with groups of between 15 and 20 people who were provided with information about the study and instructions on how to answer the questions. Participants returned the survey to boxes located in each of the health care facilities. The surveys were collected over a 1–2 week period. The questionnaires did not contain any details which could identify the worker and, once collected, were kept in such a manner that only the research team could have access to them, thus ensuring total confidentiality. Informed consent was given by every participant prior to their inclusion in the study and the authorization of the Local Ethics Committee was obtained for every centre studied.

The information (N = 871) was analysed following the conventional definition and recommendation of the Health and Safety Executive (HSE) for Psychosocial Hazard Measures [27].

### Statistical analysis and psychometric measures

Sample size characteristics were summarized by mean and standard deviation in case of quantitative variable and by percentage in case of qualitative variable.

The descriptive analysis was compared with standard values from both questionnaires manuals. A series of t-tests contrasted the Spanish sample with norms on the nine variables in the study (three dimensions, and six sub-scales), using a criterion of α = .0056 as a Bonferroni correction for multiple comparisons. The reference scores for the MBI-GS subscales are from the MBI Manual [14]. The norms for the Areas of Worklife Scale come from it’s the manual based upon 17,079 responses to the questionnaire, encompassing 35 surveys of diverse occupations in seven languages from around the world [8].

Data on the reliability (internal consistency and reproducibility), content validity, construct validity and predictive validity were obtained. Thus, with regard to reliability, the first coefficient was calculated Cronbach α evaluating internal consistency. Whereas a good Cronbach coefficient those values above 0.7. As a minimum cut off for group comparisons, and 0.9 for individual comparisons. Subsequently, we calculated the intra-class correlation coefficient (ICC), which measures the test-retest reproducibility. The ICC ranges from = (no agreement) to 1 (perfect agreement), and a value greater than 0.75 is considered excellent agreement. The reproducibility of the questionnaire was evaluated in the same way by comparing the average in the two administrations of the questionnaire (Student t test for dependent variables).

In order to study the validity of content is collected the results of factor analysis, and compared the dimensions obtained with the original questionnaire. Moreover, construct validity was assessed by correlation coefficient, and finally the predictive validity was analysed using linear regression models.

For the analysis of data was used SPSS 15.0, and were considered as statistically significant p-values all <0.05.

To study the construct validity, results of factor analysis using principal components analysis with VARIMAX rotation were obtained. In addition, a confirmatory factor analysis (CFA) it was used to test whether the factorial structure was consistent. For this aim, we have estimated a structural equation analysis to evaluate the hypothesized model and it has reported the robust statistics for Chi Square [28,29], Chi Square/degrees of freedom (χ2/df), Comparative Fit Index (CFI), and Root Mean-Square Error of Approximation (RMSEA). One criterion for a good model fit is an absolute reference point of a CFI ≥ 0.900 with an excellent fit as CFI ≥ 0.950 and a RMSEA < 0.05 [26]. χ2/df is good with a value <5 and excellent with <3 [20].

Concurrent validity of AWS was analyzed for its relationship with other measures, in particular with the opposing dimensions to burnout. This analysis was performed by the non-parametric Rho Spearman correlation, since no variable was a normal behaviour. Finally, predictive validity was analyzed using linear regression models propose by the original scale authors. According to these authors, Six Areas: workload, control, reward, community, fairness and values, are predictive of opposite burnout dimension values: Energy, Efficacy, and Implication.

For data analysis was used SPSS 15.0, and Equations (EQS), with significance to p-values < 0.05.

## Results

1,200 subjects matching the most important variable representative criteria (gender, age, profession) were selected from various centers. 947 responses were obtained, of which 871 were used. 17 questionnaires were excluded as they were incomplete. 59 more questionnaires were excluded from analysis in order to ensure that the number of responses were proportionate to staff numbers at the various institutions. The exclusions were made based on order of questionnaire submission.

The proportion of respondents by profession was: (60,7% nurses and 39,2% doctors).

Table 1 shows descriptive values from Spanish questionnaires and the comparison with standard values from manuals. Both groups (Spanish and standard values) differed in all measures, but the direction of the difference varied across dimensions. The Spanish sample reported lower scores on Exhaustion and Depersonalization/Cynicism, indicating less negative experiences regarding Energy and Involvement. They also reported lower scores on Efficacy, indicating a more negative experience on the third component of burnout. For all six areas of work-life, the Spanish sample showed more positive evaluations of workload than the normative data, but reported more negative evaluations of the other five areas of work-life.

### Reliability

The scale study reports reliability for Energy, Efficacy and Implication, and also for the six sub-scales. Reliability for all scales is high (between 0.70 and 0.90), suggesting a good level of internal consistency (Table 2). Highly reliable results were obtained by means of test-retest (N = 112), taking into account and comparing the earlier results of subjects who used the same code (Table 3). This analysis was performed with Intraclass Correlation Coefficient, that in all dimensions was higher than 0.75 and T-Student was not significative (so, means are similar in both moments).

**Table 1:** Main characteristic description from AWL in the Spanish version

	**Norms**		**Spain**			
**Measure**	**Mean**	**S.D.**	**Mean**	**S.D.**	**t**	**p-value**
Exhaustion	2.95	1.56	1.98	1.36	26.36	<0.001
Cynicism	1.80	1.30	1.64	1.31	5.84	<0.001
Efficacy	4.41	1.02	3.77	1.00	21.29	<0.001
Manageable	2.75	0.75	3.06	.83	9.36	<0.001
Control	3.08	0.78	2.73	.91	-4.45	<0.001
Reward	3.10	0.94	3.00	.82	-6.68	<0.001
Community	3.46	0.83	3.19	.82	-8.07	<0.001
Fairness	2.75	0.77	2.54	.72	-8.93	<0.001
Values	3.23	0.66	3.01	.70	-	<0.001

Comparison with norms.

### Validity analysis

The development of AWS suggests that it probably has reasonable face validity. It is the result of thirty years work on areas in work-life that can contribute to levels of energy, implication and efficiency, and it incorporates proved elements from other theories, as was shown in the description. The authors have conducted studies with samples from diverse working sectors [11], where its content validity is contrasted.

#### Construct validity

Construct validity were analyzed through Confirmatory Factor Analysis (CFA), which confirmed the structure obtained from the original version [11]. Table 4 shows factor loadings obtained from this analysis where CFA assigned the 29 items of the six sub-scales: workload, control, reward, community, fairness, and values. We can observe a perfect relation between items and the corresponding dimension.

The first item in each scale was set at 1.00. All factor covariances were freed; no error covariances were freed. As with the CFA, the value 1.00 was assigned to the first item within each construct to set the scale and all correlations among the factors were freed. Whereas some items showed a moderate kurtosis, the analysis used the robust analysis option of EQS, which corrects for multivariate kurtosis [21].

**Table 2:** Reliabilities for each dimension from the AWS(N = 871)

**Dimensions**	**Cronbach alpha coefficient**
Energy	.85
Efficacy	.83
Implication	.81
Workload	.89
Control	.79
Rewards	.80
Community	.73
Fair	.72
Values	.71

**Table 3:** Test-retest analysis

**Dimensions**	**ICC**	**Mean (test)**	**DT (test)**	**Mean (retest)**	**DT (retest)**	p-value (*)
Energy	.89	1.98	1.36	1.95	1.29	0.898
Efficacy	.80	3.77	1.00	3.79	0.89	0.857
Implication	.81	1.64	1.31	1.59	1.28	0.578
Workload	.76	3.06	.83	3.12	0.80	0.658
Control	.77	2.73	.91	2.35	0.92	0.447
Rewards	.76	3.00	.82	3.10	0.85	0.778
Community	.81	3.19	.82	3.18	0.77	0.953
Fair	.79	2.54	.72	2.59	0.83	0.388
Values	.78	3.01	.70	2.98	0.75	0.720

Intraclass Correlation Coefficient (ICC) N = 112.

* T-Student for paired sample.

The CFA produced a fit (χ^2^_(362)_ = 1292.78, *p* < .001; χ^2^/df = 3.57; CFI = 0.867; RMSEA = 0.055) much better than a One Factor Model that assigned all items to a single factor (χ^2^_(377)_ = 3600.95, *p* < 0.001; χ^2^/df = 9.55; CFI = 0.541; RMSEA = 0.103). A Two Factor Model (Factor 1: Workload; Factor 2: Other Areas) produced an improved fit (χ^2^_(376)_ = 2598.64, *p* < 0.001; χ^2^/df = 6.91; CFI = 0.684, RMSEA = 0.083) that still fell short of that of the Six Factor Model. Although all item coefficients were significant on the assigned factor, the modification indices indicated significant error correlations, especially on sequential items, for example Reward 3 with Reward 4. A modified model freed the 10 largest item’s error correlations, all of which had modification indices greater than 40 (modified Six-Factor model) produced a good fit χ^2^_(352)_ = 806.21, *p* < 0.001; χ^2^/df = 2.29; CFI = 0.935, RMSEA = 0.039).

**Table 4:** Principal components factor analysis (PCFA) of Spanish version of AWS

	**Fair**	**Workload**	**Community**	**Reward**	**Values**	**Control**
Fair 5	**–0.71**	0.08	-0.11	-0.21	0.01	0.22
Fair 6	**0.70**	0.07	-0.14	-0.21	-0.07	0.19
Fair 4	**0.68**	-0.06	0.11	0.17	0.27	0.06
Fair 3	**0.64**	-0.18	0.15	0.04	0.19	0.20
Fair 2	**0.57**	-0.04	0.02	-0.06	0.10	0.22
Fair 1	**0.53**	0.04	0.06	-0.02	0.19	0.23
Workload 4	–0.04	**0.79**	-.09	**-**0.14	-0.08	0.00
Workload 1	–0.08	**0.79**	-.015	**-**0.04	**-**0.04	-0.12
Workload 2	–0.06	**0.75**	-.01	**-**0.02	0.01	0.06
Workload 3	–0.11	**0.73**	-.02	**-**0.26	-0.07	-0.07
Workload 5	–0.02	**-0.62**	0.16	0.03	0.04	0.16
Workload 6	–0.17	**-0.55**	0.04	**-**0.02	0.18	-0.15
Community 3	0.13	**-**0.10	**0.82**	0.12	0.09	-0.02
Community 4	0.16	**-**0.08	**0.82**	0.09	0.06	0.09
Community 1	0.11	**-**0.07	**0.73**	0.06	0.08	0.10
Community 5	0.04	0.11	**-0.53**	-0.20	-0.02	0.01
Community 2	0.07	-0.07	**0.47**	0.21	0.05	0.04
Rewards 4	-0.18	0.13	**-**0.10	-**0.81**	-0.08	-0.02
Rewards 3	-0.11	0.20	**-**0.13	-**0.79**	-0.10	0.00
Rewards 1	0.21	-0.05	0.23	**0.62**	0.21	0.29
Rewards 2	-0.03	0.05	0.27	**0.58**	0.10	0.18
Values 1	0.20	-0.10	0.06	0.12	**0.75**	-0.01
Values 1	0.22	-0.09	0.08	0.21	**0.73**	0.03
Values 1	0.16	0.03	0.05	**-**0.02	**0.70**	0.14
Values	-0.14	0.16	-0.20	**-**0.12	**-0.56**	-0.02
Values	0.27	-0.16	0.10	0.17	**0.53**	0.13
Control 1	0.15	-0.14	0.00	0.19	0.00	**0.72**
Control 3	0.40	0.03	0.18	0.23	0.18	**0.55**
Control 2	0.12	-0.16	0.21	0.29	**-**0.12	**0.46**

* Factor loadings obtenidas al realizar el analisis factorial de componentes principales.

Using modification indices to free error correlations among items presents the risk of improving the fit arbitrarily. We justify the procedure here because: 1) the modifications are limited to items within the same factor; 2) the modifications were limited to error correlations for sequential items, and 3) only 10 of the 406 error correlations among the 29 items were freed. The presences of correlated errors between sequential items in questionnaires are likely to reflect response sets. These limitations minimize the modifications’ implications for the factor structure the testing of which was the primary objective of this analysis.

A principal components analysis of the normative sample provided evidence supporting a six-factor structure for the AWS (percentage of variance explained by the six-factor was 73.45%): The scree plot determined that Eigen values began levelling after six factors: 7.64, 2.53, 1.83, 1.60, 1.33, and 1.24. Two items had loadings that were less than ∣0.50: Community 2 loaded on Community 0.47 with a second highest loading of 0.21 on Rewards. Control 2 loaded on Control at 0.46 with a second highest loading of 0.29 on Rewards. As the second loadings for both items were considerably lower than the loading on the proper factor, the overall structure is acceptable.

#### Concurrent validity

The concurrent validity of AWS was analysed for its relationship with other measures, in particular with the opposing dimensions to burnout, as contemplated in the model: Energy, Implication and Efficacy (Table 5); our results were similar (MBI-GS) to those used in the Spanish validation [8] (Table 6). Each correlation between dimensions and sub-dimensions were statistically significant.

#### Predictive validity

For this aim, a series of multiple regression analysis examined the resulting patterns of the CFA further. The outcome variables were, on one hand, the positive dimensions which Leiter y Maslach proposed for burnout: Energy, Implication, and Efficacy (Table 7), and on the other hand the three dimensions of MBI: Emotional Exhaustion, Depersonalization (or Cynicism), and Personal Accomplishment (Table 8).

**Table 5:** Spearman’s correlations

	**Workload**	**Control**	**Reward**	**Community**	**Fair**	**Values**
Energy	0.48	0.45	0.45	0.41	0.28	0.37
Efficacy	0.12	0.23	0.30	0.27	0.12	0.17
Implication	0.26	0.29	0.38	0.34	0.19	0.29

Positive dimension of burnout with AWS.

All correlations are significant at the 0.01 level (2-tailed).

First, the analysis confirms the relationships between Six Areas with the three dimensions of burnout, and with the three positive dimensions of burnout (Table 7). The Six Areas (except fairness: t = -1,03; p = 0,301; B = -0,80)) explained the 65% variance in Energy. In Implication, all areas were statistically significant and they explained the 50% in variance and finally, the 35% variance in Efficacy was explained by four of six sub-scales. Workload (t = 0.012, p ≤ 0.990; B = 0,00) and value (t = 1.463, p = 0.144; B = 0,05) were not in the model.

In regards to the MBI dimensions (see Table 8); six Areas (except fairness: t = -0.08, p = 0.936; B = -0.01) explained the 71% variation. In Depersonalization (or Cynicism), areas explained the 41% variance being all variables statistically significant except for control and the 37% variance Personal Accomplishment was explained by.

### Mediation model

**Figure 2:**
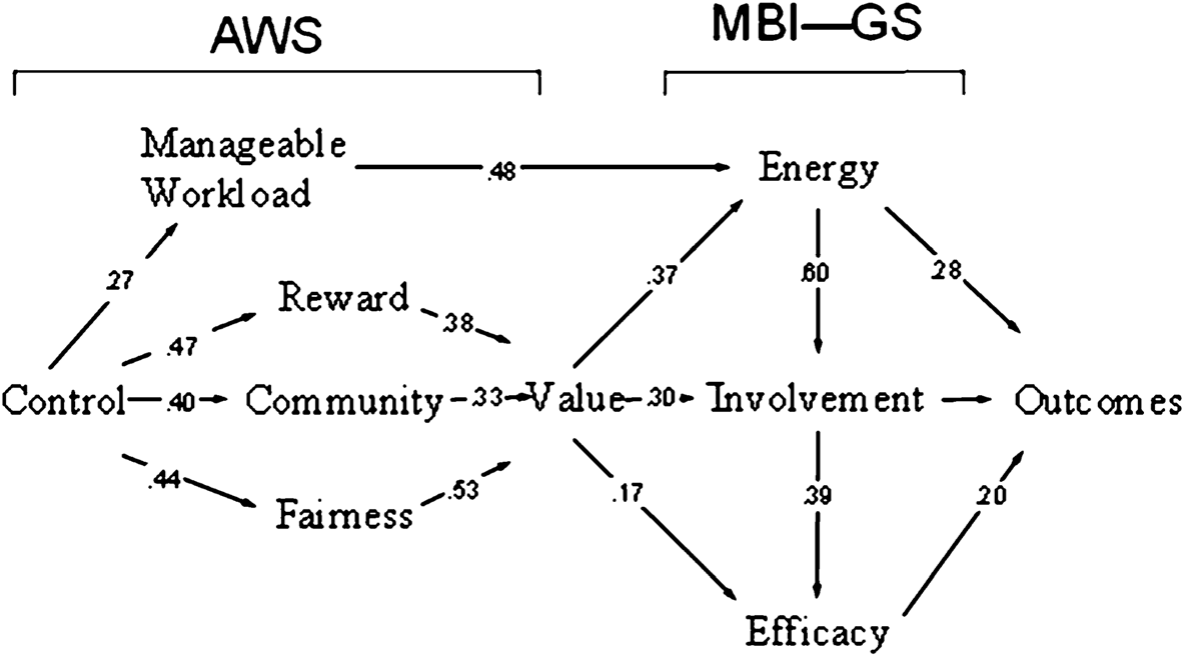
The SEM of Spanish version of AWL.

The Structural Equation Model (SEM) used the AWS as defined in the CFA. For the MBI-GS, the analysis included three indicators for each of the three subscales as defined in Leiter et al [11], permitting the analysis to focus primarily on the structural relationships among the constructs and to deemphasize the factor structure of the MBI-GS that has been established elsewhere [8,22]. The Mediated Model produced a weak fit regarding the CFI, but an adequate fit regarding the χ^2^/df and RMSEA (χ^2^_(642)_ = 1683.27, *p* < 0.001; χ^2^/df = 2.62; CFI = 0.895, RMSEA = 0.044). The modification indices identified three areas of worklife for which values did not fully mediate their relationships with the burnout aspects: Reward with Exhaustion, Community with Cynicism, and Reward with Efficay. Adding these three paths to a Partially Mediated Model provided a better fit (χ^2^_(639)_ = 1588.90, *p* < 0.001; χ^2^/df = 2.49; CFI = 0.904, RMSEA = 0.042) that was a significant improvement over the Mediated Model (χ^2^[3] = 94.37, *p* < 0.001) and bringing the CFI to an adequate level. Figure 2 show the SEM of Spanish version of AWL.

## Discussion

The translation of the English version of the AWS questionnaire into Spanish showed good psychometric properties.

Reliability for all scales was high (between 0.71 and 0.90), suggesting a good level of internal consistency, and highly reliable results were obtained by means of test-retest (N = 112), taking into account and comparing the earlier results of subjects who used the same code. By Intraclass Correlation Coefficient was higher than 0.75 in all dimensions and T-Student was not significative (so, means are similar in both moments).

**Table 6:** Spearman’s correlation

	**Workload**	**Control**	**Reward**	**Community**	**Fair**	**Values**
emotional exhaustion	-0.63	-0.36	-0.43	-0.37	-0.30	-0.42
depersonalization	-0.29^*^	-0.25	-0.29	-0.30	-0.19	-0.29
personal accomplishment	0.14	0.22	0.30	0.29	0.08	0.19

MBI dimensions, AWS Sub-Scales.

All correlations are significant at the 0.01 level (2-tailed).

About to validity, a Confirmatory Factor Analysis confirmed the structure obtained from the original version [11] in six sub-scales: workload, control, reward, community, fairness, and values; as well as we can observe in the relation between items and the corresponding dimension. The percentage of variance explained by the six-factor was 73.45%.

Concurrent validity of AWS was observed for its relationship with other measures: MBI variables (Exhaustion, Depersonalization, and Personal Accomplisment, and with opposing dimensions to burnout (Energy, Implication and Efficacy) [30].

In 2000, Leiter & Maslach developed a new questionnaire on the extent of burnout [9-11], which is based on its previous MBI [8], with the difference that measures the opposite dimensions. Ie, instead of Emotional Exhaustion, provides an index of Energy, and instead of Depersonalization, provides an idex of Involvement. The Personal Accomplishment dimension or sense of Efficacy remains positive dimension in both questionnaires. The difference with the MBI is that some of the items are made in reverse, or inverted scores should be taken once the results [9]. Therefore, the dimensions of both questionnaires obtained high correlations to be compared. But the big news of this questionnaire is that it adds a scale of six areas of worklife that may be contributing positively or negatively in the dimensions between burnout and engagement. These areas are: manageable overload, control, rewards, community, fairness, and values [11]. In different studies, the six dimensional structure or the questionnaire has been confirmed, and its contribution to predicting the three main variables by regression analysis [9,31].

**Table 7:** Regression analysis

	**Coefficient**	**Std. error**	**T-student**	**p-valor**
**Dependent variable energy. R = 0.646**				
Constant	2.51	1.48	1.69	0.091
workload	0.54	0.06	11.89	<0.001
control	0.54	0.12	4.58	<0.001
reward	0.56	0.10	5.61	<0.001
Community	0.33	0.07	4.36	<0.001
values	0.42	0.09	4.41	<0.001
**Dependent variable implication. R = 0.498**				
Constant	6.27	0.75	8.41	<0.001
workload	0.09	0.03	3.04	0.002
control	0.13	0.06	2.20	0.028
reward	0.29	0.05	5.92	<0.001
Community	0.18	0.04	4.88	<0.001
fair	-0.10	0.04	2.51	0.012
values	0.23	0.05	4.91	<0.001
**Dependent variable efficacy. R = 0.355**				
Constant	5.59	0.62	9.12	<0.001
control	0.15	0.05	3.15	0.012
reward	0.17	0.04	4.27	<0.001
Community	0.13	0.03	4.28	<0.001
fair	-0.08	0.03	2.55	0.011

Positive Dimension of Burnout.

All correlation between dimensions and sub-dimensions were statistically significant.

**Table 8:** Regression analysis: MBI factors

	**B**	**Std. error**	**t**	**p-valor**
**Dependent variable: emotional exhaustion. R = 0.711**				
Constant	54.45	1.39	39.14	<0.001
workload	-1.01	0.05	-19.23	<0.001
control	-0.23	0.11	-2.03	0.042
reward	-0.52	0.09	-5.52	<0.001
Community	-0.20	0.07	-2.86	0.004
values	-0.46	0.09	-5.08	<0.001
**Dependent variable: depersonalisation (Cynicism). R = 0.415**				
Constant	15.15	0.74	20.33	<0.001
workload	-0.14	0.03	-5.15	<0.001
control	-0.08	0.06	-1.32	0.187
reward	-0.13	0.05	-2.52	0.012
Community	-0.16	0.04	-4.22	<0.001
fair	0.02	0.04	-0.61	0.542
values	-0.16	0.05	-3.37	0.001
**Dependent variable: personal accomplishment. R = 0.370**				
Constant	16.14	1.34	12.02	<0.001
workload	0.04	0.05	0.86	0.390
control	0.23	0.11	2.14	0.033
reward	0.39	0.09	4.38	<0.001
Community	0.33	0.07	4.93	<0.001
fair	0.19	0.07	0.38	0.001
values	0.19	0.09	2.29	0.022

For know the predictive validity, a series of multiple regression analysis examined the resulting patterns of the CFA further. Taking into account as outcome variables, those already mentioned in the previous paragraph. This analysis confirms the relationships between Six Areas with the three dimensions of burnout, and with the three positive dimensions of burnout.

These results are consistent with those obtained in the validation into other languages, for example a confirmatory factor analysis assessed the extent to which the factor structure of the English version of the scale transferred to a Finnish translation [31] with four samples of workers: healthcare, education, postal workers and telecommunications. Also, a confirmatory factor analysis assessed the extent to which the factor structure of the English version of the scale transferred to an Italian translation [11] with samples of health care workers.

These analyses provide strong evidence for the utility of the Areas o Worklife Scale (AWS) as a means of assessing organizational life, which is a key factor in the mediation model. The scale produces a consistent factor structure, defining six areas of worklife of specific relevance to the continuum from burnout to engagement as assessed by the MBI-GS [11].

As a weak point to consider: while it is true that the fact is using modification indices to free error correlations among items presents the risk of improving the fit arbitrarily. We have already justified the procedure because: the modifications are limited to items within the same factor; the modifications were limited to error correlations for sequential items, and only 10 of the 406 error correlations among the 29 items were freed. The presences of correlated errors between sequential items in questionnaires are likelty to reflect response sets. These limitations minimize the modifications’ implications for the factor structure the testing of which was the primary objective of this analysis.

We believe that AWS marks an important development in the design of hazard measures. It is designed not only to explain how the individual scores in respect to Energy, Implication and Efficacy, but also how the variables in work-life influence these dimensions. The Leiter and Maslach theoretical framework is based on the assumption that burnout is not an individual problem, but an imbalance among certain areas of work-life and the employee [10]. On one hand, if an organization, or a concrete sector, demonstrates strength in an area, or has a problem affecting any of the dimensions of burnout, it would be more effective to act on the organization rather than on particular individuals [9]. On the other hand, seeing a clear diagram of the areas which positively or negatively affect the dimensions of burnout makes it easy for employees to form groups to implement change, oriented by professionals, to propose and apply measures which may contribute to positive change.

The questionnaire is not addressed to individuals that we consider “unwell”, but towards a global vision of change in the organization which promotes a culture of permanent prevention. Its ease of use enables anybody working in prevention to implement the evaluation and, with the help of professionals, produce a report on the situation, in order to design prevention policies, capitalizing on strong aspects and acting on troublesome areas.

Even though the subjects of the present study have been health professionals, as they are more exposed to burnout than other workers [32,33], the questionnaire can be utilized in other organizations [25].

The survey showed evidence of two processes, as has been seen in previous research – the workload/exhaustion process and the values/burnout process-, and defines burnout as something more than an exhaustion syndrome [16]. The results also supported a six factor model for the Spanish translation of the AWS. Firstly, work overload exhausts employees by exerting excessive demands and interfering with their energy recovery capacity. Secondly, enduring personal and organizational value conflicts have a diverse relationship with burnout.

Often, professionals are unsure of how to detect and prevent burnout, because they lack awareness of available burnout assessment tools as well as effective prevention methods. In order to design good intervention programs, there is a clear need for accurate instruments to evaluate and detect burnout [33].

Research on the role of value congruence, in building work engagement and preventing burnout has the potential for making a major contribution to this field. An element of a research agenda is to consider factors that shape employees’ career values. This exploration can take into account the origin of long-term career narratives from a developmental perspective. It will also examine the development of professional values through the process of advanced education. Another element is the process through which employees determine organizational values. One element of this process will be the employees’ familiarity with the organizations’ official mission statements, vision, and corporate values. A parallel process includes employees’ evaluation of the values implicit in decisions and priorities evident in day-to-day organizational life.

The analysis presented above regarding relationships of areas of work-life with value congruence demonstrates, in a general way, the relevance that work experience has on employees’ judgments about values. A more detailed examination of the cognitive and emotional processes underlying these judgments would provide a deeper perspective on how work-life experience influences employees’ experience of burnout or work engagement.

A more ambitious research agenda would consider interventions to enhance value congruence, assessing their impact over time. Potential interventions could target corporate communication as well as the responsiveness of organizations to employees’ values. Regarding corporate communication, interventions could consider ways in which organizations state their values and their procedures to assure that these values are a major part of decisions. Employees are likely to perceive value incongruence when they notice that the organization as taking action that contradicts the organization’s stated values. This incongruity may occur due to poor communication -employees fail to understand the strategies underlying these events- or because of weak management controls -managers making important decisions without reference to organizational values. The first problem could benefit from interventions that focus on improving communication from managers while the second problem could be addressed by better communications with managers from executive levels of the organization. The choice of intervention would depend on an organizational assessment. The research could evaluate the impact of these interventions by examining changes in the communication as well as evaluating employees’ perceptions of value congruence over time. Another intervention pathway is developing processes through which organizations become more aware of and responsive to employees’ values. An increased capacity for senior management to listen to employees and consider the values employees bring to their work has the potential to develop a more engaging work setting.

In summary, the AWS is a good instrument for assessing relationships between employees and their organizations that relate to the three dimensions of burnout. The AWS provides an important tool for assessing quality of worklife in organizations as well as designing intervention programs. It also provides an excellent tool for the early prediction of burnout.

It is important to point out that it is not an instrument for individual evaluation, but to evaluate dimensions opposing burnout –Energy, Implication, Efficacy-, and all other variables which contribute positively or negatively to burnout: manageable workload, control, possibility, community feeling, intrinsic rewards, coherence of values and a sense of fairness.

The questionnaire offers an exhaustive evaluation. It is very easy to implement, by professionals or employees themselves. With the resulting chart, a team consisting of professionals in prevention issues, along with the company’s employees can study the situation and suggest measures for improvement, drawing on the strong aspects of the organization and trying to act on the areas with highest amount of risk. Obviously it is necessary to evaluate during and after process, in order to ensure that the tool is being managed correctly and improvements are applied in the future. This method of working, not only contributes to the prevention of psycho-social risks, but also creates a culture of permanent prevention and well-being in the organization [34,35].

Research projects of great interest are being carried out nowadays, not only in the area of health, but also in the fields of education and other public sector areas, in Spain, México and Argentina. These projects have some factors in common: they have all performed a previous evaluation using AWS, and, once the results were analyzed, they designed preventative policies with company management: establishing positive two-way communication channels between management and employees; elaborating fair reward systems (professional career, permanent training, research opportunities) and clear appeal systems in cases of perceived injustice; offering support to professionals who have suffered aggression in the form of, medical, psychological and legal attention, as well as striving to prevent these violent incidences, etc.

The potential interest of this AWS translation is enormous for places like Spain or Latin American countries, where there is no clear legislation regarding psycho-social risks.

## Competing interest

The authors declare that they have no competing interests.

## Authors’ contributions

SG, MPL, MAS, JMM and JG-C conceptualised the study. SG, ASM and NS wrote the manuscript, JMM and EA carried out the statistical analysis and all authors participated in critically revising for important intellectual content and have given final approval of the version to be published. All authors read and approved the final manuscript.

## References

[B1] Schaufeli WB Leiter MP Maslach C Burnout: 35 years of research and practice Career Dev Int 2009 14 3 204 220 10.1108/13620430910966406

[B2] Martínez De Viergol A La consideración del síndrome de burnout como constitutivo de la contingencia profesional de Accidente de Trabajo Revista del Ministerio de Trabajo y Asuntos Sociales 2005 59 213 224

[B3] Work Risk Prevention Law Ley 31/1995 de 8 de noviembre de Prevención de Riesgos Laborales BOE n° 269 10/11/1995

[B4] Gascón S Intervención sobre riesgos psicosociales: una asignatura pendiente 2006 Available from: https://www.prevencionintegral.com/.

[B5] Cherniss C Professional burnout in human service organizations 1980 New York: Praeger

[B6] Freudenberger HJ Staff burnout J Soc Issues 1974 30 159 165

[B7] Bakker AB Demerouti E Schaufeli WB Validation of the Maslach Burnout Inventory General Survey: An Internet study Anxiety Stress Coping 2002 15 245 260 10.1080/1061580021000020716

[B8] Maslach C Jackson SE Leiter MP Maslach Burnout Inventory Manual 1986 3 Palo Alto, CA: Consulting Psychologists Press

[B9] Leiter MP Maslach C Preventing burnout and building engagement: A training package 2000 San Francisco: Jossey Bass

[B10] Leiter MP Maslach C S**ix Areas of worklife: A model of the organizational context of burnout** J Health Hum Resour Adm 1999 21 472 489 10621016

[B11] Leiter MP Maslach C Ganster , Perrewé Areas of worklife: A structured approach to organizational predictors of job burnout Research in occupational stress and well being. Emotional and physiological processes and positive intervention strategies 2004 3 Oxford, UK: JAI Press/Elsevier 91 134

[B12] Maslach C Schaufeli WB Leiter MP Job Burnout Annu Rev Psychol 2001 52 397 422 10.1146/annurev.psych.52.1.397 11148311

[B13] Halbesleben JR Buckley MR Burnout in organizational life J Manage 2004 30 859 879

[B14] Seisdedos N Manual MBl, Inventario Burnout de Maslach 1997 Madrid: TEA

[B15] Leiter MP Maslach C The Areas of Worklife Scale Manual 2002 Wolfville, NS, Canada: Centre for Organizational Research & Development

[B16] Leiter MP Gascón S Martínez-Jarreta B A Two Process Model of Burnout: Their Relevance to Spanish and Canadian Nurses Psychol Spain 2008 12 1 37 45

[B17] Maslach C Leiter MP The truth about burnout 1997 San Francisco: Jossey Bass

[B18] Karasek R Theorell T Stress, productivity, and the reconstruction of working life 1990 New York: Basic Books

[B19] Landsbergis PA Occupational stress among health care workers: A test of the job demands-control model J Org Behav 1988 9 3 217 239 10.1002/job.4030090303

[B20] Bowers L Nijman H Simpson A Jones J The relationship between leadership, teamworking, structure, burnout and attitude to patients on acute psychiatric wards Soc Psychiatry Psychiatr Epidemiol 2011 46 2 143 148 10.1007/s00127-010-0180-8 20082064 PMC3034905

[B21] Cordes CL Dougherty TW A review and integration of research on job burnout Acad Manage Rev 1993 18 621 656

[B22] Leiter MP Gascón S Martínez-Jarreta B Making sense of worklife: a structural model of burnout J Appl Soc Psychol 2010 40 57 75 10.1111/j.1559-1816.2009.00563.x

[B23] Reininghaus U Priebe S Assessing morale in community mental health professionals: a pooled analysis of data from four European countries Soc Psychiatry Psychiatr Epidemiol 2007 42 3 237 243 10.1007/s00127-007-0154-7 17268760

[B24] Walsh WB, Craik KH, Price RH Person-environmental psychology: Models and perspectives 1992 Hillsdale NJ: Lawrence Erlbaum

[B25] Montero-Marín J García-Campayo J A newer and broader definition of burnout: Validation of the “Burnout Clinical Subtype Questionnaire (BCSQ-36)” BMC Public Health 2010 10 302 10.1186/1471-2458-10-302 20525178 PMC2887826

[B26] Aaronson NK Acquadro C Alonso J Apolone G Bucquet D Bullinger M International Quality of Life Assessment (IQOLA) Project Qual Life Res 1992 1 349 351 10.1007/BF00434949 1299467

[B27] Rick J Briner RB Daniels K Perryman S Guppy A A critical review of psychosocial hazard measures 2001 U.K: Health & Safety Executive 65 75

[B28] Satorra A Bentler PM Scaling correction for chi-square statistics in covariance structure analysis 1988 North Washington Street, Alexandria: Proceeding of the American Statistical Association 308 313

[B29] Byrne B Structural equation modelling with EQS and EQS/Windows: Basic concepts, applications and programming 1994 Thousand Oaks, CA: Sage

[B30] Vercambre MN Brosselin P Gilbert F Nerrière E Kovess-Masféty V Individual and contextual covariates of burnout: a cross-sectional nationwide study of French teachers BMC Public Health 2009 9 333 10.1186/1471-2458-9-333 19744328 PMC2754990

[B31] Aro A Kärná P Salmela-Aro Leiter MP Maslach C Development of the Finís version of Areas of Worklife Survey. Unpublished data 2001 Universty of Helsinki

[B32] Montero-Marín J García-Campayo K Fajó-Pascual M Carrasco JM Gascón S Gili M Mayoral-Cleries F Sociodemographic and accupational risk factors associated with the development of different burnout types: the cross-sectional University of Zaragoza Study BMC Psychiatry 2011 11 1 49 10.1186/1471-244X-11-49 21447169 PMC3074532

[B33] Kahn RL Byosiere P Dunnette MD, Hough LM Stress in organizations Handbook of Industrial and Organizational Psychology 1992 3 571 650

[B34] Uher R Goodman R The everyday Feeling Questionnaire: the structure an validation of measure psychological well-being and distress Soc Psychiatry Psychiatr Epidemiol 2010 45 3 413 423 10.1007/s00127-009-0074-9 19466369

[B35] Bossarte RM He H Claassen CA Knox K Tu X Development and validation of a 6-day standard for the identification of frequent mental distress Soc Psychiatry Psychiatr Epidemiol 2011 46 5 403 411 10.1007/s00127-010-0204-4 20401465

